# The value of NT-proBNP, NLR, Ang-1 combined with APACHE II and SOFA scores in evaluating 28-day mortality of septic shock

**DOI:** 10.1097/MD.0000000000042547

**Published:** 2025-06-06

**Authors:** Longping Peng, Xiangyi Yuan, Lele Li, Jueting Liu, Jian Ma, Shan Zhong

**Affiliations:** a Orthopaedics, Bayi Orthopaedics Hospital, Chengdu, Sichuan, China.

**Keywords:** acute physiology and chronic health evaluation II, angiopoietin-1, neutrophil-to-lymphocyte ratio, N-terminal pro-B-type natriuretic peptide, septic shock, sequential organ failure assessment

## Abstract

This study investigates the value of combining N-terminal pro-B-type natriuretic peptide (NT-proBNP), neutrophil-to-lymphocyte ratio (NLR), angiopoietin-1 (Ang-1) with the acute physiology and chronic health evaluation II (APACHE II) and sequential organ failure assessment (SOFA) scores for the assessment of 28-day mortality in septic shock. This retrospective study enrolled 121 hospitalized patients with septic shock admitted from February 2022 to February 2023 and followed them up. They were grouped based on whether they died within 28 days. 44 patients who died were included in the death group, while the other 77 patients were included in the survival group. Clinical data differences between the groups were compared, and the predictive value of NT-proBNP, NLR, Ang-1, APACHE II, and SOFA scores for mortality within 28 days was explored. The patients in the death group and the survival group showed no differences in gender, past medical history (hypertension, diabetes, cerebrovascular disease, and chronic obstructive pulmonary disease), recent surgical history, age, shock index, and oxygenation index (*P* > .05). However, the levels of blood creatinine and lactate were higher in the death group compared to the survival group (*P* < .05). The levels of NT-proBNP, NLR, Ang-1, as well as APACHE II and SOFA scores were higher in the deceased group compared to the survival group (*P* < .05). Multiple-factor logistic regression analysis indicated that creatinine, NT-proBNP, NLR, Ang-1, APACHE II score, and SOFA score all significantly influenced the survival status of patients (*P* < .05). Based on the aforementioned multifactorial analysis results, a logistic regression model was established. Using this model as the independent variable, ROC curve analysis was conducted with patient mortality within 28 days as the dependent variable. The area under the curve (AUC) value was found to be 0.844, with a 95% CI of 0.772 to 0.916. Sensitivity was 0.841, and specificity was 0.701. The optimal cutoff value was determined to be 123.56. The model exhibited good fit (Hosmer-Lemeshow χ2 = 3.458, *P* = .788). The combined analysis of NT-proBNP, NLR, Ang-1 levels, along with APACHE II and SOFA scores, can effectively predict the 28-day mortality risk in patients with septic shock. These indicators have important clinical value in early identification of high-risk patients and guiding treatment decisions.

## 1. Introduction

Sepsis shock is one of the most challenging clinical syndromes in intensive care medicine, with its high mortality imposing a heavy burden on patients’ families and society as a whole.^[1]^ Timely and accurate risk assessment is crucial for the treatment and prognosis of septic shock patients. In recent years, the academic community has been continuously exploring and validating the application value of various biomarkers and scoring systems in prognostic assessment of septic shock. N-terminal pro-B-type natriuretic peptide (NT-proBNP) is a sensitive marker of cardiac stress and cardiac dysfunction. Recent studies have found significant changes in its levels in noncardiac diseases, especially in severe infections and shock.^[2]^ The neutrophil-to-lymphocyte ratio (NLR), reflecting inflammation and immune response, has been proven to have prognostic predictive value in various clinical conditions.^[3]^ Angiopoietin-1 (Ang-1), as a vascular stabilizing factor, its level changes are closely related to alterations in vascular permeability and inflammatory response in patients with septic shock.^[4]^ The acute physiology and chronic health evaluation II (APACHE II) and sequential organ failure assessment (SOFA) scores are commonly used tools to assess the severity of illness and organ dysfunction in critically ill patients. They play an important role in predicting the prognosis of patients with septic shock.^[5,6]^ Although the aforementioned indicators are closely associated with the progression of septic shock, further exploration is needed to determine whether they can accurately assess the prognosis of septic shock. Based on this, the aim of this study is to integrate these biomarkers and scoring systems to investigate their combined application in predicting the 28-day mortality risk of patients with septic shock. This is intended to provide clinicians with a more comprehensive and precise assessment tool, thereby improving treatment strategies and prognosis for patients with septic shock.

## 2. Materials and methods

### 2.1. General data

This retrospective study was approved by the Ethics Committee of Bayi Orthopaedic Hospital. This study enrolled 121 hospitalized patients with septic shock admitted from February 2022 to February 2023 and followed them up, stratifying them based on whether they died within 28 days. Among them, 44 deceased patients were included in the death group, while the remaining 77 patients were included in the survival group. This study was approved by the hospital’s ethics committee.

Inclusion criteria: (1) All patients met the relevant diagnostic criteria in the International Guidelines for the Management of Sepsis and Septic Shock^[7]^: there were clear infection lesions, and the SOFA score was higher than 2 points, and fluid replacement was required to maintain normal vital signs; (2) First-diagnosed patients; (3) age ≥ 18 years old; and (4) The clinical data were complete and available for study.

Exclusion criteria: (1) Immunosuppressive therapy was used within 3 months before enrollment; (2) cardiac insufficiency caused by coronary heart disease or other heart diseases; (3) liver and kidney dysfunction; and (4) autoimmune deficiency.

### 2.2. Observation indicators and acquisition methods

All data were collected for the first time when patients were admitted to hospital.

Clinical data collection: Clinical data was collected through reviewing medical records, including gender, age, presence of hypertension, diabetes, cerebrovascular disease, oxygen saturation, and recent surgical history.

Shock index: Using an intensive care monitor to detect pulse rate and systolic blood pressure, take 3 consecutive measurements and calculate the average. Shock index = pulse rate/systolic blood pressure.

Biochemical index detection: Collect 5 mL of fasting venous blood from the patient, then centrifuge at a speed of 4200 r/min for 10 minutes with a centrifugal radius of 10 cm. Subsequently, use the electrochemiluminescence immunoassay method to detect serum NT-proBNP. Use the XN-1000 hematology analyzer produced by Sysmex Corporation to detect levels of neutrophils and lymphocytes. Blood creatinine (CRE) and blood lactate (Lac) are measured using the Bc-7500 fully automatic biochemical analyzer produced by Mindray Medical International Limited. Ang-1 is determined by enzyme-linked immunosorbent assay, with the reagent kit selected from Abbott Laboratories, Abbott Park. NLR = Neutrophil count/Lymphocyte count.

APACHE score^[8]^: The Acute Physiology and Chronic Health Evaluation score is composed of acute physiology score, age score, and chronic health condition score. Theoretically, the highest score can reach 71 points. A higher score indicates a more severe condition of the patient.

SOFA score^[9]^: It covers 6 organ systems, including the respiratory system, coagulation system, liver function, cardiovascular system, central nervous system, and renal function. The score for each system ranges from 0 (normal) to 4 (most severe dysfunction). The total score of the SOFA score is obtained by adding the scores of these 6 systems. A higher total score indicates a more severe degree of organ failure in the patient, with a worse prognosis.

### 2.3. Statistical processing

In this study, SPSS 26.0 software was used to analyze and sort out the data. The count data collected in this study were tested by chi-square test. The measurement data were in line with normal distribution and homogeneity of variance. Independent sample t test was performed between groups. The factors affecting the death of patients within 28 days were explored by multivariate logistic regression analysis, and the predictive value was analyzed by ROC curve. Test level α = 0.05.

## 3. Results

### 3.1. Comparison of clinical data

The gender, past medical history (hypertension, diabetes, cerebrovascular disease, and chronic obstructive pulmonary disease), recent surgical history, age, shock index, and oxygenation index were not significantly different between the death group and the survival group (*P* > .05). However, the levels of blood creatinine and blood lactate were higher in the death group compared to the survival group (*P* < .05). See Table 1.

**Table 1:** Comparison of clinical data.

Items	Death group (n = 44)		Survival group (n = 77)		*χ*2	*P*
	Number of cases	Proportion (%)	Number of cases	Proportion (%)		
Sex						
Male	20	45.45	41	53.25	0.680	.410
Female	24	54.55	36	46.75		
Hypertension						
Yes	23	52.27	45	58.44	0.433	.511
No	21	47.73	32	41.56		
Diabetes						
Yes	16	36.36	19	24.68	1.861	.173
No	28	63.64	58	75.32		
Cerebrovascular disease						
Yes	8	18.18	16	20.78	0.119	.730
No	36	81.82	61	79.22		
Chronic obstructive pulmonary disease						
Yes	10	22.73	15	19.48	0.180	.671
No	34	77.27	62	80.52		
Recent history of surgery						
Yes	5	11.36	8	10.39	0.028	.868
No	39	88.64	69	89.61		
Items		Death group (n = 44)		Survival group (n = 77)	*t*	*P*
Age (years old)		68.15 ± 13.02		71.11 ± 12.02	−1.264	.209
Shock index		1.35 ± 0.12		1.37 ± 0.16	−0.721	.472
Oxygenation index		266.46 ± 41.02		254.45 ± 38.46	1.613	.109
Serum creatinine(μmol/L)		133.26 ± 22.11		98.41 ± 16.46	9.862	.000
Blood lactic acid (mmol/L)		4.22 ± 0.87		2.03 ± 0.56	16.836	.000

### 3.2. Comparison of NT-proBNP, NLR and Ang-1 levels

The levels of NT-proBNP, NLR, and Ang-1 were all higher in the death group compared to the survival group among patients (*P* < .05). See Table 2.

**Table 2:** Comparison of NT-proBNP, NLR and Ang-1 levels.

Items	NT-proBNP (ng/mL)	NLR	Ang-1 (ng/mL)
Death group (n = 44)	8686.16 ± 1225.46	15.65 ± 2.13	40.26 ± 3.11
Survival group (n = 77)	987.21 ± 123.61	10.36 ± 2.98	34.12 ± 2.98
*t*	54.813	10.353	10.731
*P*	.000	.000	.000

Ang-1 = Angiopoietin-1, NLR = neutrophil-to-lymphocyte ratio, NT-proBNP = N-terminal pro-B-type natriuretic peptide.

### 3.3. Comparison of APACHE II score and SOFA score

Patients in the death group had higher APACHE II scores and SOFA scores compared to those in the survival group (*P* < .05). See Table 3.

**Table 3:** Comparison of APACHE II score and SOFA score.

Item	APACHE II score (score)	SOFA score (score)
Death group (n = 44)	24.55 ± 3.02	10.68 ± 1.06
Survival group (n = 77)	21.02 ± 2.45	9.11 ± 1.12
*t*	6.996	7.561
*P*	.000	.000

APACHE II = Acute physiology and chronic health evaluation II, SOFA = Sequential organ failure assessment.

### 3.4. Multivariate logistic regression analysis of 28-day mortality in patients

Using whether the patient died within 28 days as the dependent variable (1 for yes, 0 for no), multiple logistic regression analysis was conducted on the patients’ levels of blood creatinine, blood lactate, NT-proBNP, NLR, Ang-1, APACHE II score, and SOFA score (all measured values). The results, as shown in Table 4, indicate that blood creatinine, NT-proBNP, NLR, Ang-1, APACHE II score, and SOFA score all significantly influence the survival status of patients (*P* < .05). See Table 4.

**Table 4:** Multivariate logistic regression analysis of 28-day mortality in patients.

Factor	B	SE	Wald	*P*	OR	95%CI	
						Lower limit	Upper limit
Serum creatinine	0.106	0.02	28.373	.000	1.112	1.069	1.156
Blood lactic acid	0.011	1.046	0.131	.995	1.007	0.130	7.823
NT-proBNP	0.147	1.616	14.469	.000	7.495	1.685	12.564
NLR	0.789	0.148	28.441	.000	2.202	1.647	2.943
Ang-1	0.711	0.142	25.153	.000	2.036	1.542	2.688
APACHE II score	0.461	0.09	26.049	.000	1.585	1.328	1.892
SOFA score	1.278	0.244	27.502	.000	3.590	2.226	5.787

Ang-1 = angiopoietin-1, APACHE II = acute physiology and chronic health evaluation II, NLR = neutrophil-to-lymphocyte ratio, NT-proBNP = N-terminal pro-B-type natriuretic peptide, SOFA = sequential organ failure assessment.

### 3.5. Establishment of a predictive model for 28-day mortality of patients

Based on the results of the multiple factor analysis, the logistic regression model was established with whether the patient died within 28 days as the outcome variable. For variables with differences, the model equation is as follows: $P=\frac{e^{logit(\;p)}}{1+e^{logit(\;p)}}$. Where logit(P) = 0.106 × Blood Creatinine + 0.147 × NT-proBNP + 0.789 × NLR + 0.711 × Ang-1 + 0.461 × APACHE II Score + 1.278 × SOFA Score.

### 3.6. The predictive value of the predictive model for 28-day death of patients

**Figure 1.**
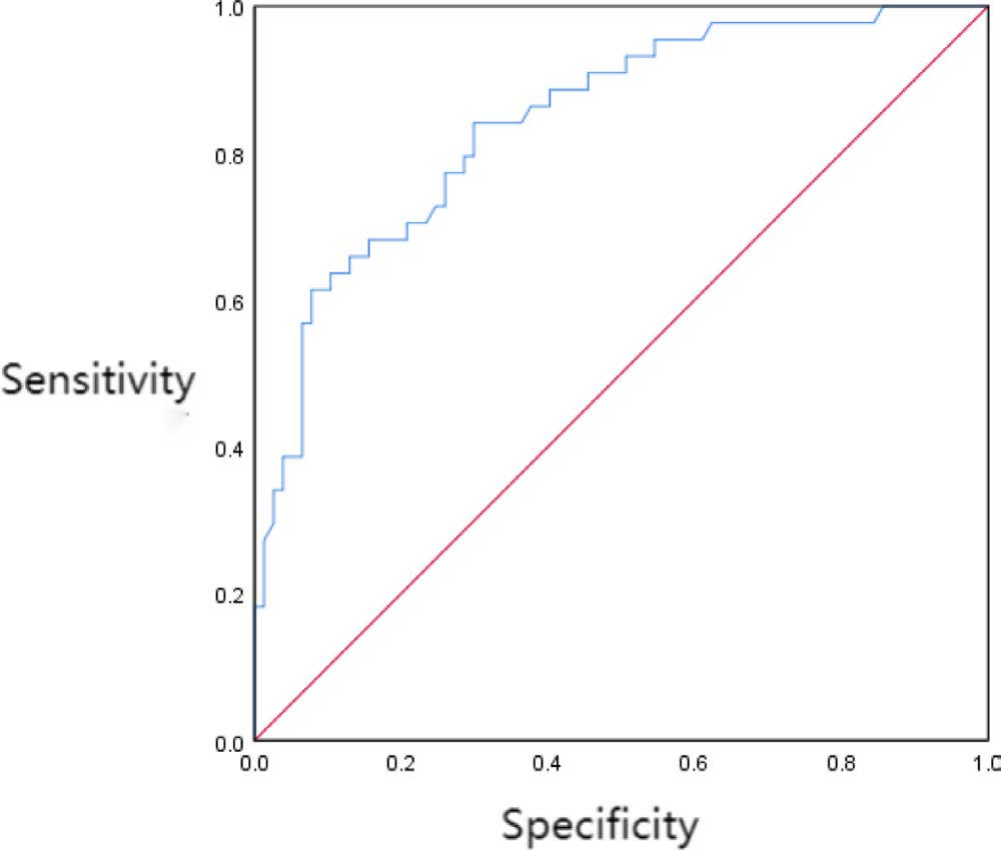
The predictive value of the predictive model for 28-day death of patients.

When each factor is entered into the corresponding predictive model, with its *P*-value as the independent variable and whether the patient died within 28 days as the dependent variable, ROC curve analysis yielded an AUC value of 0.844, with a 95% confidence interval of 0.772 to 0.916. The sensitivity was 0.841, and the specificity was 0.701. The optimal cutoff value was determined to be 123.56. The model demonstrated good fit (Hosmer-Lemeshow χ2 = 3.458, *P* = .788). See Figure 1.

## 4. Discussion

Infectious shock has always been a significant challenge in the field of clinical medicine due to its high mortality rate and poor prognosis.^[10–12]^ The pathogenesis of infectious shock is complex, involving various aspects such as host inflammatory response, immune dysfunction, endotoxin release, and microcirculatory disorders. Therefore, there are diverse factors influencing the prognosis of patients with infectious shock, including age, underlying diseases, cause and severity of infection, organ function status, timeliness and effectiveness of treatment measures, etc.^[13,14]^ Research indicates that the mortality risk of patients with infectious shock is closely related to the levels of various biomarkers. For example, NT-proBNP, as a marker of cardiac stress, its elevation can reflect the degree of cardiac dysfunction; NLR, as an indicator reflecting the body’s inflammatory status and immune function, its changes may indicate the patient’s inflammatory response and immune status; Ang-1, as a key factor in vascular stability and repair, its decrease may be associated with increased vascular permeability and aggravated inflammatory damage in patients with infectious shock. In addition, comprehensive scoring systems such as APACHE II and SOFA scores provide clinicians with quantitative tools to assess the severity of a patient’s condition and prognosis by evaluating physiological parameters and organ function. The application of these scoring systems helps physicians comprehensively assess the condition of patients with septic shock, guiding treatment decisions and optimizing resource allocation. However, a single indicator may not fully reflect the patient’s condition and prognosis. Combining biomarkers such as NT-proBNP, NLR, Ang-1, with comprehensive scoring systems like APACHE II and SOFA may offer a more comprehensive and accurate risk assessment.

This study first analyzed the clinical data of 2 groups of patients. The results showed no significant differences between the death group and the survival group in terms of gender, past medical history (such as hypertension, diabetes, cerebrovascular disease, and chronic obstructive pulmonary disease), recent surgical history, age, shock index, and oxygenation index. This suggests that these characteristics do not significantly affect the risk of death in the 2 groups of patients. Additionally, compared to the survival group, patients in the death group showed significantly elevated levels of blood creatinine and blood lactate, indicating that renal dysfunction and tissue hypoxia may be associated with an increased risk of death in patients with septic shock. The study results also revealed that serum levels of NT-proBNP, NLR, Ang-1, as well as APACHE II and SOFA scores, were significantly higher in the death group compared to the survival group. Further logistic regression analysis indicated that creatinine, NT-proBNP, NLR, Ang-1, APACHE II, and SOFA scores were all factors influencing the 28-day mortality of patients with septic shock.

In septic shock, elevated blood creatinine levels typically indicate the presence of acute kidney injury, which is not only a common complication of septic shock but also an important indicator of disease severity and poor prognosis.^[15]^ Moreover, the elevation in blood creatinine levels may also affect the function of other organs, as renal dysfunction can lead to the accumulation of toxins, electrolyte imbalance, and disturbances in acid-base balance, thereby affecting the function of vital organs such as the heart, lungs, and brain. This multi-organ dysfunction further exacerbates the patient’s condition and increases the risk of mortality.^[16–18]^ In a state of cardiac stress, ventricular myocardial cells secrete NT-proBNP, and its increased levels are associated with cardiac dilation and impaired myocardial function. The occurrence of hypotension and tissue hypoperfusion in septic shock can exacerbate cardiac burden, leading to impaired cardiac function. NT-proBNP, as a marker of cardiac dysfunction, reflects the degree of cardiac damage and is closely related to patient prognosis.^[19,20]^ Alataby et al^[21]^ through their research, found that elevated levels of serum lactate, cardiac troponin, and NT-proBNP are independent predictors of mortality in patients with sepsis and septic shock when assessing prognosis. This is consistent with the results of this study.

NLR is an effective marker for assessing the severity of infection, reflecting the body’s stress response to severe infections. It is characterized by a significant increase in neutrophil count and a decrease in lymphocyte count, resulting in elevated NLR. Specifically, in the context of septic shock, the body’s immune system is extensively activated. Neutrophils, serving as the primary immune defense line, rapidly respond to microbial invasion detection, with their numbers sharply increasing. They play a crucial role in the immune response by directly engulfing pathogens, releasing various pro-inflammatory cytokines, and activating T cells.^[22,23]^ This intense immune response, while beneficial in combating pathogens, can also lead to excessive tissue damage, thereby triggering multi-organ failure or death. Additionally, the body releases a large quantity of anti-inflammatory cytokines, resulting in lymphocyte apoptosis. In septic shock, neutrophil apoptosis may also be delayed, further increasing the number of immature neutrophils in circulation.^[24–26]^

Ang-1 is a critical growth factor in the human body, typically expressed at low levels under normal physiological conditions. However, during inflammation, particularly in the case of septic shock, levels of Ang-1 significantly increase. Ang-1 specifically binds to receptors on the surface of endothelial cells, leading to an imbalance in intracellular signaling and subsequently affecting the stability of vascular function. In septic shock, this imbalance exacerbates vascular dysfunction, further impacting patient prognosis. Specifically, the elevation of Ang-1 during the inflammatory response exacerbates vascular permeability and the degree of inflammation in patients with septic shock. These changes not only worsen the condition but may also lead to multiple organ dysfunction, increasing the risk of mortality.^[27–29]^ Therefore, the levels of Ang-1 are considered an important biomarker for assessing the prognosis of patients with septic shock.

The APACHE II scoring system comprises acute physiology score, age score, and chronic health score, comprehensively considering the patient’s physiological status, age, and preexisting health conditions. This comprehensiveness allows the APACHE II score to more accurately reflect the severity of the patient’s condition and the risk of mortality.^[4,30,31]^ The SOFA score assesses the function of 6 organ systems, including respiratory, coagulation, liver, cardiovascular, central nervous, and renal systems. It provides clinicians with a comprehensive view of the degree of organ dysfunction in patients. The extent of organ dysfunction is an important factor in predicting the prognosis of critically ill patients.^[32–34]^

Based on the multifactorial analysis results mentioned above, this study established a logistic regression model. The predictive model exhibited good performance, with high values for AUC, sensitivity, and specificity. This indicates that the model can serve as an effective tool in clinical practice for assessing the 28-day mortality risk in patients with septic shock.

This study evaluated the value of NT-proBNP, NLR, Ang-1 combined with APACHE II and SOFA scores in predicting 28-day mortality in septic shock, but there are still some limitations. First, the 28-day mortality as an endpoint does not reflect long-term survival, functional recovery, and the impact of chronic complications. Future studies should consider extending the follow-up period (such as 90 days or 6 months) for a more comprehensive prognostic assessment. Second, this study used biomarkers and scoring data from a single time point and did not consider their dynamic changes. The condition of septic shock patients often changes rapidly, and dynamic assessment of biomarkers may better reflect disease progression and treatment response. Future research will include dynamic monitoring of biomarkers and scores, evaluating the impact of changes in these indicators over time on prognostic predictions. Moreover, although Ang-1 is of significant importance in prognostic evaluation, its clinical detection is not widely applied. Future studies should explore faster and more cost-effective detection methods (such as POCT). The calculation of APACHE II and SOFA scores is relatively complex and may affect the timeliness of clinical decision-making. Future studies could enable automatic score calculation via electronic health record systems or introduce artificial intelligence to optimize prediction models. Additionally, while serum creatinine, as a marker of kidney dysfunction, has significant predictive value, this study only explored its predictive ability and did not assess the effects of specific kidney protection strategies. Future research could incorporate different kidney protection interventions to further explore their impact on prognosis. In summary, future research should combine dynamic monitoring of biomarkers, long-term follow-up, optimized detection technologies, and treatment strategies to further improve risk assessment and individualized treatment for septic shock patients.

In summary, the combination of NT-proBNP, NLR, Ang-1 levels, along with APACHE II and SOFA scores, can effectively predict the 28-day mortality risk in patients with septic shock. These indicators hold important clinical value for the early identification of high-risk patients and guiding treatment decisions.

## Author contributions

**Conceptualization:** Longping Peng, Xiangyi Yuan, Shan Zhong.

**Data curation:** Longping Peng, Xiangyi Yuan, Lele Li, Shan Zhong.

**Formal analysis:** Longping Peng, Xiangyi Yuan, Shan Zhong.

**Investigation:** Lele Li.

**Methodology:** Lele Li, Jueting Liu.

**Supervision:** Jian Ma, Jueting Liu.

**Validation:** Jian Ma.

**Writing – original draft:** Longping Peng, Shan Zhong.

**Writing – review & editing:** Longping Peng, Shan Zhong.

## References

[1] Spaggiari V Passini E Crestani S. Neonatal septic shock, a focus on first line interventions. Acta Biomed. 2022;93:e2022141. 35775767 10.23750/abm.v93i3.12577 PMC9335427

[2] Martín-Rodríguez F Melero-Guijarro L Ortega GJ. Combination of prehospital NT-proBNP with qSOFA and NEWS to predict sepsis and sepsis-related mortality. Dis Markers. 2022;2022:5351137. 35242244 10.1155/2022/5351137 PMC8886755

[3] Schupp T Weidner K Rusnak J. The neutrophil-to-lymphocyte-ratio as diagnostic and prognostic tool in sepsis and septic shock. Clin Lab. 2023;69:25–7. 10.7754/Clin.Lab.2022.220812 37145065

[4] Garcia B Su F Manicone F. Angiotensin 1-7 in an experimental septic shock model. Crit Care. 2023;27:106. 36915144 10.1186/s13054-023-04396-8 PMC10010236

[5] Lanspa MJ Cirulis MM Wiley BM. Right ventricular dysfunction in early sepsis and septic shock. Chest. 2021;159:1055–63. 33068615 10.1016/j.chest.2020.09.274 PMC7965651

[6] Hou H Yang J Han Z Zhang X Tang X Chen T. Predictive values of the SOFA score and procalcitonin for septic shock after percutaneous nephrolithotomy. Urolithiasis. 2022;50:729–35. 36214882 10.1007/s00240-022-01366-7 PMC9584975

[7] Critical Care Medicine Branch of Chinese Medical Association. Chinese guidelines for the treatment of severe sepsis/ septic shock (2014). Chin Emerg Med Crit Illn. 2015;27:401–26.

[8] Godinjak A Iglica A Rama A. Predictive value of SAPS II and APACHE II scoring systems for patient outcome in a medical intensive care unit. Acta Med Acad. 2016;45:97–103. 28000485 10.5644/ama2006-124.165

[9] Lambden S Laterre PF Levy MM Francois B. The SOFA score-development, utility and challenges of accurate assessment in clinical trials. Crit Care. 2019;23:374. 31775846 10.1186/s13054-019-2663-7 PMC6880479

[10] De Backer D Ricottilli F Ospina-Tascón GA. Septic shock: a microcirculation disease. Curr Opin Anaesthesiol. 2021;34:85–91. 33577205 10.1097/ACO.0000000000000957

[11] Fu X Lin X Seery S. Speckle-tracking echocardiography for detecting myocardial dysfunction in sepsis and septic shock patients: a single emergency department study. World J Emerg Med. 2022;13:175–81. 35646207 10.5847/wjem.j.1920-8642.2022.057 PMC9108915

[12] Foster DM Kellum JA. Endotoxic septic shock: diagnosis and treatment. Int J Mol Sci. 2023;24:16185. 38003374 10.3390/ijms242216185 PMC10671446

[13] Ranjit S Kissoon N Argent A. Haemodynamic support for paediatric septic shock: a global perspective. Lancet Child Adolesc Health. 2023;7:588–98. 37354910 10.1016/S2352-4642(23)00103-7

[14] Hellman T Uusalo P Järvisalo MJ. Renal replacement techniques in septic shock. Int J Mol Sci. 2021;22:10238. 34638575 10.3390/ijms221910238 PMC8508758

[15] Fu S Yu W Fu Q Xu Z Zhang S Liang T-B. Prognostic value of APTT combined with fibrinogen and creatinine in predicting 28-day mortality in patients with septic shock caused by acute enteric perforation. BMC Surg. 2023;23:274. 37700315 10.1186/s12893-023-02165-6 PMC10498602

[16] Borensztajn DM Tan CD de Rijke Y. Elevated high-sensitivity troponin and NT-proBNP values in febrile children. Pediatr Emerg Care. 2024;40:108–13. 38113471 10.1097/PEC.0000000000003097 PMC11444364

[17] Yoon SY Kim JS Jeong KH Kim S-K. Acute kidney injury: biomarker-guided diagnosis and management. Medicina (Kaunas). 2022;58:340. 35334515 10.3390/medicina58030340 PMC8953384

[18] Pickkers P Darmon M Hoste E. Acute kidney injury in the critically ill: an updated review on pathophysiology and management. Intensive Care Med. 2021;47:835–50. 34213593 10.1007/s00134-021-06454-7 PMC8249842

[19] Wang J Dong Y Zhao B Liu K. Preoperative NT-proBNP and LVEF for the prediction of acute kidney injury after noncardiac surgery: a single-centre retrospective study. BMC Anesthesiol. 2022;22:196. 35751021 10.1186/s12871-022-01727-0 PMC9229082

[20] Liang P Yu F. Predictive value of procalcitonin and neutrophil-to-lymphocyte ratio variations for bloodstream infection with septic shock. Med Sci Monit. 2022;28:e935966. 35509186 10.12659/MSM.935966 PMC9083214

[21] Alataby H Nfonoyim J Diaz K. The levels of lactate, troponin, and N-terminal Pro-B-type natriuretic peptide are predictors of mortality in patients with sepsis and septic shock: a retrospective cohort study. Med Sci Monit Basic Res. 2021;27:e927834. 33518698 10.12659/MSMBR.927834 PMC7863562

[22] Su L Zhang J Gomez H Kellum JA Peng Z. Mitochondria ROS and mitophagy in acute kidney injury. Autophagy. 2023;19:401–14. 35678504 10.1080/15548627.2022.2084862 PMC9851232

[23] Juan CX Mao Y Cao Q. Exosome-mediated pyroptosis of miR-93-TXNIP-NLRP3 leads to functional difference between M1 and M2 macrophages in sepsis-induced acute kidney injury. J Cell Mol Med. 2021;25:4786–99. 33745232 10.1111/jcmm.16449 PMC8107088

[24] Botoș ID Pantiș C Bodolea C. The dynamics of the neutrophil-to-lymphocyte and platelet-to-lymphocyte ratios predict progression to septic shock and death in patients with prolonged intensive care unit stay. Medicina (Kaunas). 2022;59:32. 36676656 10.3390/medicina59010032 PMC9861709

[25] Martin-Sanchez D Guerrero-Mauvecin J Fontecha-Barriuso M. Bone marrow-derived RIPK3 mediates kidney inflammation in acute kidney injury. J Am Soc Nephrol. 2022;33:357–73. 35046131 10.1681/ASN.2021030383 PMC8819996

[26] Yan YT Liu HM Kong YF. Association of preoperative neutrophil-lymphocyte ratio with acute kidney injury in patients with non-cardiac surgery: difference among surgical types. Int Urol Nephrol. 2023;55:2647–56. 36964822 10.1007/s11255-023-03567-4

[27] Drăgoescu AN Pădureanu V Stănculescu AD. Neutrophil to Lymphocyte Ratio (NLR)-a useful tool for the prognosis of sepsis in the ICU. Biomedicines. 2021;10:75. 35052755 10.3390/biomedicines10010075 PMC8772781

[28] Akatsuka M Tatsumi H Sonoda T Masuda Y. Low immunoglobulin G level is associated with poor outcomes in patients with sepsis and septic shock. J Microbiol Immunol Infect. 2021;54:728–32. 32859530 10.1016/j.jmii.2020.08.013

[29] Wei RL Li ZF Cao RS. New analysis of Banxia Xiexin Decoction and its similar prescriptions in Treatise on Febrile Diseases to explore the universality of the treatment and compatibility principle. World J Tradit Chin Med. 2022;8:509–13.

[30] Hu X Ning X Zhao Q. Islet-1 mesenchymal stem cells-derived exosome-incorporated angiogenin-1 hydrogel for enhanced acute myocardial infarction therapy. ACS Appl Mater Interfaces. 2022;14:36289–303. 35920579 10.1021/acsami.2c04686

[31] Bloria SD Chauhan R Sarna R Gombar S Jindal S. Comparison of APACHE II and APACHE IV score as predictors of mortality in patients with septic shock in intensive care unit: a prospective observational study. J Anaesthesiol Clin Pharmacol. 2023;39:355–9. 38025575 10.4103/joacp.joacp_380_21 PMC10661619

[32] Li Q Chai W Wang X. Epidemiological analysis of septic shock in the plateau region of China. Front Med (Lausanne). 2022;9:968133. 36186819 10.3389/fmed.2022.968133 PMC9515411

[33] Jacobi J. The pathophysiology of sepsis - 2021 update: part 2, organ dysfunction and assessment. Am J Health Syst Pharm. 2022;79:424–36. 34651652 10.1093/ajhp/zxab393

[34] Balamuth F Scott HF Weiss SL. Validation of the pediatric sequential organ failure assessment score and evaluation of third international consensus definitions for sepsis and septic shock definitions in the pediatric emergency department. JAMA Pediatr. 2022;176:672–8. 35575803 10.1001/jamapediatrics.2022.1301 PMC9112137

